# Evaluation of the public health empowerment program in the Eastern Mediterranean region

**DOI:** 10.3389/fpubh.2023.1180678

**Published:** 2023-05-26

**Authors:** Majd A. Alsoukhni, Mohannad Al Nsour, Ruba Kamal Alsouri, Abdulwahed Al Serouri, Zakir Hussain, Labiba Saeed Annam, Abdulhakeem Al Kohlani, Mumtaz Ali Khan, Sahar Mahmoud Samy, Nissaf Bouafif ép Ben Alaya, Ilham B. Abu-Khader, Haitham Bashier Abbas, Yousef Khader

**Affiliations:** ^1^Center of Excellence for Applied Epidemiology, Global Health Development, Eastern Mediterranean Public Health Network, Amman, Jordan; ^2^Global Health Development, Eastern Mediterranean Public Health Network, Amman, Jordan; ^3^Yemen Field Epidemiology Training program (FETP), Ministry of Public Health and Population, Sana'a, Yemen; ^4^National Institute of Health, Islamabad, Pakistan; ^5^Ministry of Health and Population, Minya, Egypt; ^6^National Observatory of New and Emerging Diseases, Ministry of Health, Tunis, Tunisia; ^7^Department of Community Medicine, Public Health, and Family Medicine, Faculty of Medicine, Jordan University of Science & Technology, Irbid, Jordan

**Keywords:** field epidemiology, public health, outbreak, surveillance, evaluation

## Abstract

**Background:**

The Public Health Empowerment Program (PHEP) is a 3-month training program for frontline public health staff to improve surveillance quality and strengthen the early warning system capacities. Studies evaluating the program and its impact on the health systems in the Eastern Mediterranean Region (EMR) are lacking. Therefore, this study aimed to assess the level of PHEP graduates’ engagement in field epidemiology activities, assess their perceived skills and capacity to perform these activities and assess the extent to which PHEP helped the graduates to perform field epidemiology activities.

**Methods:**

A descriptive evaluation study was conducted based on levels 3 and 4 of Kirkpatrick’s model for evaluating training programs to assess the change in graduates’ behavior and the direct results of the program. Data were collected using two online surveys targeting PHEP graduates and programs’ directors/ technical advisers.

**Results:**

A total of 162 PHEP graduates and 8 directors/ technical advisers participated in the study. The majority of PHEP graduates reported that they are often involved in activities such as responding to disease outbreaks effectively (87.7%) and monitoring surveillance data collection (75.3%). High proportions of PHEP graduates rated their skills as good in performing most of field epidemiology activities. The majority of graduates reported that the PHEP helped them much in conducting, reviewing, and monitoring surveillance data collection (92%), responding effectively to public health events and disease outbreaks (91.4%), and communicating information effectively with agency staff and with the local community (85.2%).

**Conclusion:**

PHEP appears to be an effective program for improving the public health workforce’s skills and practices in epidemiological competencies in the EMR. PHEP strengthened the engagement of the graduates in most field epidemiology activities, especially during COVID-19.

## Background

Many countries in the Eastern Mediterranean Region (EMR) have been affected by various types of disasters including war, political conflicts and instabilities, massive forced displacement, and natural disasters (1). These emergencies have exposed many countries to increased public health threats and affected the health security of the entire region (2, 3). Furthermore, these emergencies, together with the COVID-19 pandemic, boosted the demand for training programs to provide public health professionals with a diverse range of skills required to improve global health security (4, 5).

The Public Health Empowerment Program (PHEP) is a frontline Field Epidemiology Training Program that was introduced to enhance global health security by training frontline public health staff to improve surveillance quality and strengthen the early warning system capacities in their districts (6). In 2015, the US Centers for Disease Control and Prevention (CDC) launched this three-month in-service training program in 24 countries to enhance local public health capacity (7). The program focuses on the detection and response to diseases and other public health events of national and international concerns. It is intended for Ministry of Health (MOH) staff responsible for surveillance, data collection, compilation, reporting, and response at the local health system level. The main goal of this program is to build the epidemiologic capacity, strengthen public health surveillance and promote the use of data for decision-making at sub-national levels in stable and challenging conditions (8). Through PHEP, the participants learn and practice fundamental skills used in surveillance, outbreak investigation, and basic management such as basic epidemiology, case definition, disease detection and reporting, interpretation and presentation of data, case investigation and response, surveillance monitoring and evaluation, and analysis of data for use in decision-making (8).

In the EMR, the Eastern Mediterranean Public Health Network (EMPHNET) works in partnership with MOHs to develop the training curriculum (9). The program’s participants attend three workshops and complete field projects to practice, implement, and reinforce what they have learned under the supervision and support of their field mentors. By the end of the fieldwork, participants write and submit a report that describes their field experience and the newly gained and/or improved skills (8). Participants who complete the program receive a certificate of completion signed by MOH and EMPHNET.

EMPHNET has been actively working in multiple countries across the EMR, including Yemen, Oman, Iraq, Egypt, Sudan, Tunisia, Lebanon, Morocco, Jordan, Saudi Arabia, and Pakistan. The primary focus of the efforts has been to establish and implement FETPs of different modalities, while also providing frontline health workers and surveillance officers with the necessary training to prevent and respond to disease epidemics. These countries identified and expressed a need for public health professionals who possess the skills and expertise necessary to combat infectious diseases. The implementation of PHEP is crucial as it builds capacity, strengthens disease surveillance, enables early detection and response, promotes effective public health interventions, and contributes to the development of sustainable health systems. Based on the public health needs of the country, EMPHNET worked on providing one or more of the four customized designs of the program including PHEP-Basic Field Epidemiology (PHEP-BFE), PHEP-Surveillance Polio Officers (PHEP- SPO), and PHEP – Nutrition, and PHEP-Water. To date, a total of 1,303 participants graduated from the program from the 11 mentioned countries and 658 of those graduates were trained by EMPHNET.

Periodic evaluations of PHEP are necessary to maintain high-quality training, ensure that the program has achieved its aim and objectives (10), and enable countries to track the effectiveness of the programs in detecting and responding to emergencies (7). Internationally, some studies evaluated advanced FETPs and reported the experiences and lessons learned (11–13). However, only a few studies evaluated frontline PHEP (7, 14). One study was conducted to describe the process and early results of the implementation of Frontline FETP worldwide (7), which showed that FETP-Frontline can be a valuable strategy to strengthen public health capacity and enhance global health security by improving surveillance quality. Moreover, it was found that this program helped different countries to rapidly detect, respond to, and contain public health emergencies at the source. In Kenya, one study that evaluated the impact of the 3-month frontline FETP for local public health workers showed that 68% of respondents acquired new epidemiological skills and 83% applied those skills to their day-to-day work (14).

For different country programs in the EMR, there were no studies evaluating these programs. Therefore, this study aimed to assess the level of PHEP graduates’ engagement in field epidemiology activities, assess their perceived skills and capacity to perform these activities and assess the extent to which PHEP helped the graduates to perform field epidemiology activities. EMPHNET conducted this evaluation as a systematic assessment to measure how well programs’ goals and objectives are met as perceived by PHEP graduates in the region.

## Methods

A descriptive study was conducted to evaluate the PHEP in the EMR. We used levels 3 and 4 of Kirkpatrick’s model for evaluating the training (15). Level 3 of this model is about behavior “the degree to which participants apply what they learned during training when they are back on the job.” Level 4 is about results “the degree to which targeted outcomes occur as a result of the training and the support and accountability package.”

Two separate online questionnaires were developed using “CrowdSignal” tool. The first questionnaire (Supplementary File) was delivered to PHEP graduates from six different countries in the EMR that implemented the program including Egypt, Iraq, Jordan, Pakistan, Yemen, and Tunisia. The first section of the questionnaire collected information on the participants’ demographic characteristics, highest educational degree earned, country and year of graduation. The other sections included questions to measure the perceived skills and behavior of PHEP graduates regarding the program competencies and the involvement of the graduates in key areas of field epidemiology. Each competency was assessed as an integrated set of knowledge, skills, and attitude. PHEP graduates were asked about their involvement and engagement in 16 field epidemiological activities, the extent to which PHEP helped them to perform specific field epidemiology activities, and their perceived skills and capacity to perform these activities. In each question, the participants were requested to choose an appropriate response on a 5 Likert scale. The questionnaire was developed in English and then translated to Arabic using the forward-backward translation method. The questionnaire was administered in Arabic and English languages according to the preference of participants. The questionnaire was pilot tested on 10 graduates and minimal changes have been made. The face validity of the tool was established by having it reviewed by three persons.

The PHEP graduates’ database that was developed by EMPHNET was used to select a random systematic sample of 200 PHEP graduates. The online questionnaire was sent by email to the selected graduates. Two reminders, a week apart, were sent to those who did not respond to the questionnaire.

The second questionnaire was developed to be filled by the programs’ directors/ technical advisers asking them about the observed impact of the program. The questionnaire included questions on general characteristics, 5-Likert scale questions, multiple-choice questions, and a few open-ended questions. The characteristics included gender, highest educational degree earned, job title, affiliation, and country. In the second part of the survey, the questions were designed to measure the impact of the program on the health system in terms of public health priorities (disease surveillance, disease outbreaks and investigation, etc.) in addition to the COVID-19 response. The questions covered the graduates’ level of engagement in field epidemiological activities, how the directors/ technical advisers evaluate the graduates’ performance and the role of PHEP graduates in responding to COVID-19. The respondents were asked to make suggestions for the improvement of the PHEP.

Data were exported to IBM SPSS (IBM Corp, Released 2016, IBM SPSS Statistics for Windows, Version 24.0, Armonk, NY: IBM Corp.) for analysis. Data were described using percentages. The frequency distributions were presented for the main three outcome variables: engagement of PHEP graduates in field epidemiology activities (often, sometimes, rarely, and never), the extent to which PHEP has helped the graduates to perform field epidemiology activities (much, somewhat, little, and never), and perceived skills and capacity of the graduates (good, acceptable, and poor). Chi-square test and binary logistic regression were used to compare the three PHEP modalities in helping the graduates to perform the basic field epidemiology activities. A value of *p* of less than 0.05 was considered statistically significant.

## Results

### Participants’ characteristics

Of all invitees, 162 (81%) PHEP graduates responded to the online survey. The responses were from 6 countries including Egypt, Iraq, Jordan, Pakistan, Yemen, and Tunisia. Almost two third (*n* = 116, 74.7%) of the respondents were enrolled in the PHEP-BFE, 13.6% (*n* = 22) in PHEP – Nutrition, and 11.7% (*n* = 19) in PHEP- SPO. The participants’ characteristics are shown in Table 1. A total of 8 directors/ technical advisers from Egypt, Iraq, Jordan, and Pakistan participated in the evaluation of the program.

**Table 1:** The characteristics of 162 public health empowerment program (PHEP) graduates.

Variable	*n*	%
Gender		
Female	30	18.5
Male	132	81.5
Age (year)		
<35	74	54.7
35–40	62	38.3
>40	26	16
Country name		
Egypt	15	9.3
Iraq	93	57.4
Jordan	16	9.9
Pakistan	20	12.3
Yemen	7	4.3
Tunisia	11	6.8
PHEP modality		
Basic Field Epidemiology (BFE)	121	74.7
Nutrition	22	13.6
Surveillance Polio Officers (SPO)	19	11.7

### Engagement of PHEP graduates in field epidemiology

Table 2 shows the extent of engagement of PHEP graduates in field epidemiology activities. The majority of PHEP graduates reported that they were often involved in field epidemiology activities such as reviewing and monitoring surveillance data (*n* = 122, 75.3%), performing descriptive data analysis (*n* = 105, 64.8%), communicating information effectively with agency staff and the local community (*n* = 129, 79.6%), and responding effectively to disease outbreaks (*n* = 142, 87.7%). Almost two thirds (*n* = 104, 64.2%) of the participants reported that they were often involved in providing support to applying isolation and infection control protocols for confirmed COVID-19 cases, and almost half of them (*n* = 75, 46.3%) were often involved in collecting samples and screening passengers for COVID-19. Moreover, almost two thirds (*n* = 111, 68.5%) of the participants were often involved in monitoring the global trends of COVID-19, managing COVID-19 surveillance data (*n* = 106, 65.4%), and dissemination of health education messages and promotional materials to raise awareness toward COVID-19 (*n* = 115, 71%). For the other activities, half of the participants were often involved in the development of national guidelines for the COVID-19 epidemic (*n* = 81, 50%).

**Table 2:** The extent of engagement of public health empowerment program graduates in field epidemiology activities.

Field epidemiology activities	Often		Sometimes		Rarely	
	*n*	%	*n*	%	*n*	%
Conduct, review, and monitor surveillance data collection	122	75.3	26	16.1	14	8.6
Perform descriptive data analysis	105	64.8	43	26.5	14	8.6
Communicate information effectively with agency staff and with the local community	129	79.6	19	11.7	14	8.6
Respond effectively to public health events, specifically, disease outbreaks	142	87.7	13	8	7	4.3
Write a summary report on surveillance findings or an outbreak investigation	110	67.9	35	21.6	17	10.5
Use Microsoft Excel or any software to enter, analyze, and display public health surveillance data	134	82.7	15	9.3	13	8
Prepare and administer an oral presentation of their fieldwork	109	67.3	37	22.8	16	9.9
Monitor the global trends of COVID-19 and mortality through relevant websites	111	68.5	39	24.1	12	7.4
Manage COVID-19 surveillance data (data analysis and reporting)	106	65.4	23	14.2	33	20.4
Contribute to the development and distribution of a standard case definition for the COVID-19	84	51.9	37	22.8	41	25.3
Provide support applying isolation and infection control protocols for confirmed COVID-19 cases	104	64.2	29	17.9	29	17.9
Collect samples and screen passengers for testing to confirm suspected COVID-19 cases	75	46.3	27	16.7	60	37
Dissemination of health education messages and promotional materials to raise awareness towards COVID-19.	115	71	25	15.4	22	13.6
Respond to public queries about COVID-19 through the specified hotlines and develop documents with standard appropriate information.	75	46.3	44	27.2	43	26.5
Search for published scientific literature, standard operating procedures, and guidelines, and support the development of national guidelines for the COVID-19 epidemic.	81	50	47	29	34	21

### Skills and capacity of PHEP graduates

Majority of the participants evaluated their skills as good in conducting most of the epidemiological activities (Table 3) such as conducting, reviewing, and monitoring surveillance data collection (*n* = 136, 84%), communicating information effectively with agency staff and the local community (*n* = 128, 79%), responding effectively to disease outbreaks (*n* = 138, 85.2%), performing descriptive data analysis (*n* = 123, 75.9%), and preparing and administering oral presentations of their fieldwork (*n* = 128, 79%). For the other activities that are related to the COVID-19 pandemic, PHEP graduates also evaluated their skills as good in conducting activities such as managing and reporting COVID-19 surveillance data (*n* = 112, 69.1%), and dissemination of health education messages and promotional materials to raise awareness toward COVID-19 (*n* = 124, 76.5%). However, only half of them thought that their skills are good in contributing to the development of a standard case definition for COVID-19 (*n* = 89, 54.9%).

**Table 3:** Field epidemiology training program graduates’ self-evaluation of their skills in performing field epidemiology activities.

Field epidemiology activities	Good		Acceptable		Poor	
	*n*	%	*n*	%	*n*	%
Conduct, review, and monitor surveillance data collection	136	84	18	11.1	8	4.9
Perform descriptive data analysis	123	75.9	28	17.3	11	6.8
Communicate information effectively with agency staff and with the local community	128	79	22	13.6	12	7.4
Respond effectively to public health events, specifically, disease outbreaks	138	85.2	19	11.7	5	3.1
Write a summary report on surveillance findings or an outbreak investigation	120	74.1	32	19.8	10	6.2
Use Microsoft Excel or any software to enter, analyze, and display public health surveillance data	130	80.2	19	11.7	13	8
Prepare and administer an oral presentation of their fieldwork	128	79	27	16.7	7	4.3
Monitor the global trends of COVID-19 and mortality through relevant websites	117	72.2	35	21.6	10	6.2
Manage COVID-19 surveillance data (data analysis and reporting)	112	69.1	37	22.8	13	8
Contribute to the development and distribution of a standard case definition for the COVID-19	89	54.9	53	32.7	20	12.3
Provide support applying isolation and infection control protocols for confirmed COVID-19 cases	106	65.4	35	21.6	21	13
Collect samples and screen passengers for testing to confirm suspected COVID-19 cases	92	56.8	37	22.8	33	20.4
Dissemination of health education messages and promotional materials to raise awareness toward COVID-19.	124	76.5	26	16	12	7.4
Respond to public queries about COVID-19 through the specified hotlines and develop documents with standard appropriate information.	103	63.6	37	22.8	22	13.6
Search for published scientific literature, standard operating procedures, and guidelines, and support the development of national guidelines for the COVID-19 epidemic.	93	57.4	46	28.4	23	14.2

### The extent to which PHEP helped the graduates to perform field epidemiology activities

The level to which PHEP helped the graduates to perform field epidemiology activities is shown in Table 4. The participants reported that PHEP helped them to a considerable (much) extent in conducting, reviewing, and monitoring surveillance data collection (*n* = 149, 92%), responding effectively to disease outbreaks (*n* = 148, 91.4%), performing descriptive data analysis (*n* = 143, 88.3%), and communicating information effectively with agency staff and with the local community (*n* = 138, 85.2%). Moreover, PHEP graduates reported that the program helped them much in writing summary reports on surveillance findings or outbreak investigations (*n* = 136, 84%), using Microsoft Excel or any software to enter, analyze, and display public health surveillance data (*n* = 133, 82.1%), and preparing and administering oral presentations of their fieldwork (*n* = 132, 81.5%).

**Table 4:** The extent to which field epidemiology training program helped the graduates to perform field epidemiology activities.

Field epidemiology activities	Much		Somewhat		Little	
	*n*	%	*n*	%	*n*	%
Conduct, review, and monitor surveillance data collection	149	92	4	2.5	9	5.5
Perform descriptive data analysis	143	88.3	9	5.5	10	6.2
Communicate information effectively with agency staff and with the local community	138	85.2	8	4.9	16	9.9
Respond effectively to public health events, specifically, disease outbreaks	148	91.4	7	4.3	7	4.3
Write a summary report on surveillance findings or an outbreak investigation	136	83.9	15	9.3	11	6.8
Use Microsoft Excel or any software to enter, analyze, and display public health surveillance data	133	82.1	20	12.3	9	5.6
Prepare and administer an oral presentation of their fieldwork	132	81.5	14	8.6	16	9.9
Monitor the global trends of COVID-19 and mortality through relevant websites	116	71.6	24	14.8	22	13.6
Manage COVID-19 surveillance data (data analysis and reporting)	124	76.5	16	9.9	22	13.6
Contribute to the development and distribution of a standard case definition for the COVID-19	119	73.5	13	8	30	18.5
Provide support in applying isolation and infection control protocols for confirmed COVID-19 cases	117	72.2	19	11.7	26	16
Collect samples and screen passengers for testing to confirm suspected COVID-19 cases	94	58	19	11.7	49	30.2
Dissemination of health education messages and promotional materials to raise awareness toward COVID-19.	126	77.8	10	6.2	26	16
Respond to public queries about COVID-19 through the specified hotlines and develop documents with standard appropriate information.	108	66.7	18	11.1	36	22.2
Search for published scientific literature, standard operating procedures, and guidelines, and support the development of national guidelines for the COVID-19 epidemic.	100	61.7	17	10.5	45	27.8

Regarding the activities that are related to the COVID-19 pandemic, almost two thirds (*n* = 124, 76.5%) of the participants reported that PHEP helped them much in managing and reporting COVID-19 surveillance data, contributing to the development and distribution of standard case definition for COVID-19 (*n* = 124, 76.5%), dissemination health education messages and promotional materials to raise awareness toward COVID-19 (*n* = 126, 77.8%), responding to public queries about COVID-19 through specified hotlines and developing documents with standard appropriate information (*n* = 108, 66.7%), and monitoring global trends of COVID-19 and mortality through relevant websites (*n* = 116, 71.6%).

The graduates from PHEP-Nutrition and PHEP-SPO were significantly less likely than the graduates from PHEP-BFE to report that the program had helped them to perform the basic field epidemiology activities related to surveillance, descriptive data analysis, communication, responding to public health threats, writing summary reports, and using Microsoft Excel. The differences were significant in univariate analysis and multivariate analysis after adjusting for age and gender (Table 5). However, the three PHEP programs did not differ significantly in the extent of helping the graduates to prepare and administer oral presentations of their fieldwork.

**Table 5:** A comparison analysis of field epidemiology training programs in helping the graduates to perform the basic field epidemiology activities.

Epidemiologic activity/type of the PHEP program*	*n*	%	*p*-Value	Adjusted OR*	95% confidence interval		*P*-value
Conduct, review, and monitor surveillance data collection			<0.001				
PHEP-BFE	117	96.7		Ref			
PHEP-Nutrition	19	86.4		0.24	0.05	1.15	0.074
PHEP-SPO	13	68.4		0.07	0.02	0.28	0.000
Perform descriptive data analysis			<0.001				
PHEP-BFE	115	95.0		Ref			
PHEP-Nutrition	17	77.3		0.19	0.05	0.71	0.014
PHEP-SPO	11	57.9		0.06	0.02	0.23	0.000
Communicate information effectively with agency staff and with the local community			<0.001				
PHEP-BFE	110	90.9		Ref			
PHEP-Nutrition	14	63.6		0.16	0.05	0.52	0.002
PHEP-SPO	14	73.7		0.22	0.06	0.83	0.026
Respond effectively to public health events, specifically, disease outbreaks			<0.001				
PHEP-BFE	117	96.7		Ref			
PHEP-Nutrition	17	77.3		0.08	0.02	0.40	0.002
PHEP-SPO	14	73.7		0.07	0.01	0.34	0.001
Write a summary report on surveillance findings or an outbreak investigation			<0.001				
PHEP-BFE	107	88.4		Ref			
PHEP-Nutrition	18	81.8		0.63	0.18	2.17	0.467
PHEP-SPO	11	57.9	0.003	0.17	0.06	0.50	0.001
Use Microsoft Excel or any software to enter, analyze, and display public health surveillance data							
PHEP-BFE	108	89.3		Ref	0.06	0.52	0.001
PHEP-Nutrition	13	59.1		0.18	0.06	0.58	0.004
PHEP-SPO	12	63.2		0.19			
Prepare and administer an oral presentation of their fieldwork			0.110				
PHEP-BFE	103	85.1		Ref			
PHEP-Nutrition	15	68.2		0.38	0.13	1.06	0.064
PHEP-SPO	14	73.7		0.49	0.16	1.52	0.217

*Adjusted for the graduates’ age and gender; PHEP; Public Health Empowerment Program; BFE, Basic Field Epidemiology; SPO, Surveillance Polio Officers.

### The impact of PHEP from the perspectives of technical advisers

Five technical advisers (62.5%) reported that PHEP graduates contributed very often to conducting, reviewing, and monitoring surveillance data collection. Four technical advisers (50%) stated that almost all PHEP graduates were involved in performing descriptive data analysis and five advisers (62.5%) reported that almost all PHEP graduates communicated information with agency staff and with the local community effectively. For outbreak investigations, seven advisers (87.5%) reported that most PHEP graduates participated in outbreak investigations and responded effectively to such events. Furthermore, six advisers (75%) stated that PHEP graduates were involved in writing summary reports on surveillance findings and outbreak investigations. According to five advisers (62.5%), none or a few PHEP graduates participated in publishing research studies.

Regarding COVID-19, all technical advisers reported that almost all PHEP graduates were involved in the response to this pandemic, and they evaluated their performance as very good to excellent. Moreover, four advisers (50%) stated that PHEP graduates helped their countries to control COVID-19 to great extent. At the screening and isolation centers, technical advisers reported that PHEP graduates were involved in filling-in surveillance forms and contacting the arrivals in the follow-up period, and screening passengers at different points of entry. Finally, for the research activities, six advisers (75%) reported that PHEP graduates were engaged in working on different operational research and documented the readiness, knowledge, attitudes, and practices of the health workforce regarding COVID-19.

### Suggestions for improvement from the perspectives of PHEP technical advisers

The technical advisers of the program provided some suggestions to improve this program in their countries. Those suggestions included training more health care providers to cope with the escalating needs in the EMR countries. They recommended increasing the number of workshops in the program to ensure the improvement of participants’ epidemiological capacities and to increase the number of trainees in these programs.

## Discussion

Previous studies evaluated FETPs and reported the experiences and lessons learned (11, 12, 16). The Council of State and Territorial Epidemiologists (CSTE) evaluated the outcomes of the first 9 years of the Applied Epidemiology Fellowship (AEF) and reported that 67% of the alumni and 79% of the mentors indicated that the program was very essential and had a positive impact on their career (11). In India, the first 7 years of its FETP were evaluated and found that 86% of the fellows acquired the seven core competencies of the program (12). Another study reporting the role of Jordan FETP in the national and regional capacity building showed that the program contributed significantly to improvements in surveillance systems, control of infectious diseases, outbreak investigations, and availability of reliable morbidity and mortality data in Jordan (16). A study in Papua New Guinea on the lessons learned from the intervention-based FETP showed the successful public health outcomes with tangible local impacts of this program (17). Also, in the United Kingdom (UK), it was found that FETP highly contributed to the development of a skilled workforce in field epidemiology (13).

Although those different studies had evaluated FETP, PHEP evaluation had received little attention. PHEP is a competency-based training in basic public health and epidemiology. Our study assessed “the degree of applying what was learned” and “the degree to which outcomes occur as a result of the training” (15). The evaluation was based on information from two sources, PHEP graduates and program advisers who are within the healthcare system at a level where they can observe the impact of this program. Most graduates and their technical advisers reported that the program had helped them to perform field epidemiology activities, especially during the COVID-19 pandemic. Additionally, the program enabled them to be engaged more in conducting, reviewing and monitoring surveillance data collection, and in responding effectively to public health events, specifically, disease outbreaks. This was most apparent during the COVID-19 pandemic. PHEP graduates were actively participating in surveillance and screening at airports and other ports of entry and communicating information effectively with agency staff and with the local community.

Our evaluation showed the effectiveness of this training in improving the skills and capacity of public health workers. Our findings support the results from other studies regarding the impact of FETPs including Frontline FETP (PHEP). In Kenya, Frontline FETP graduates acquired practical skills that enhanced data collation, analysis and reporting (14). Another cross-sectional study was conducted to evaluate the first two cohorts of FETP-Frontline in Guinea (18). The evaluation showed high levels of self-reported involvement in key activities related to data collection, analysis, and reporting by program graduates. The program supervisors as well highlighted improvements to systematic and quality case and summary reporting as a result of the FETP-Frontline program.

Graduates from PHEP-Nutrition and PHEP-SPO programs reported significantly lower levels of perceived improvement in their ability to perform basic field epidemiology activities compared to graduates from the PHEP-BFE program. These activities include surveillance, descriptive data analysis, communication, responding to public health threats, writing summary reports, and using Microsoft Excel, which are essential competencies across all three modalities. The discrepancy in perceived improvement could potentially be explained by the fact that the field training in PHEP-Nutrition and PHEP-SPO programs is specifically focused on nutrition and polio, respectively, rather than providing a more comprehensive training in basic field epidemiology activities. It is possible that the training in these programs may not have been as directly applicable to the wider range of field epidemiology activities that the graduates may encounter in their professional roles.

Our study showed the high and effective engagement of PHEP graduates in responding to COVID-19 in the EMR which reflects the success of this program in building the epidemiologic capacity for the public health workforce, improving countries’ surveillance systems, and therefore strengthening health systems. Although the technical advisers reported that PHEP graduates were engaged in working on different operational research on the readiness, knowledge, attitudes, and practices of the health workforce regarding COVID-19, none, or a few of the PHEP graduates managed to participate in publishing research articles. This decreases the visibility of many achievements and successes in public health in the EMR. Therefore, it is very essential to invest more in this program to build the capacity of the public health workforce in this area.

Our results showed that PHEP helped in building a sustainable public health response capacity and expertise. Therefore, periodic evaluation is essential to ensure that the program is achieving its intended outcomes. Such evaluation helps to achieve and maintain high-quality training and assure the program’s effectiveness in improving public health. It also allows for the exchange of experiences in managing and running the program and therefore strengthens the regional emergency response mechanism and enhances coordination between MOHs in the region.

In conclusion, PHEP appears to be an effective program for improving the public health workforce’s skills and practices in epidemiological competencies. The program strengthened the engagement of the graduates in most field epidemiology activities. PHEP is essential for building the capacity in applied epidemiology. The continuity of the program should be ensured to train more people to support countries’ responses to public health events and pandemics.

## Data availability statement

The raw data supporting the conclusions of this article will be made available by the authors, without undue reservation.

## Ethics statement

The studies involving human participants were reviewed and approved by the Institutional Review Board at Jordan University of Science and Technology. Written informed consent for participation was not required for this study in accordance with the national legislation and the institutional requirements.

## Author contributions

MAA, MA, RA, HA, and YK contributed to study design, data analysis, and writing the manuscript. MAA, RA, AAS, ZH, LA, AAK, MK, SS, NB, and IA-K contributed to data collection and revision of the manuscript. All authors have read and approved the manuscript.

## Conflict of interest

The authors declare that the research was conducted in the absence of any commercial or financial relationships that could be construed as a potential conflict of interest.

## Publisher’s note

All claims expressed in this article are solely those of the authors and do not necessarily represent those of their affiliated organizations, or those of the publisher, the editors and the reviewers. Any product that may be evaluated in this article, or claim that may be made by its manufacturer, is not guaranteed or endorsed by the publisher.

## Supplementary material

The Supplementary material for this article can be found online at: https://www.frontiersin.org/articles/10.3389/fpubh.2023.1180678/full#supplementary-material
