# Supplementary material for: Evaluation of the public health empowerment program in the Eastern Mediterranean region
Source: Front Public Health. 2023 May 26;11:1180678. doi: 10.3389/fpubh.2023.1180678 (PMC10250632; doi:10.3389/fpubh.2023.1180678)
Supplement: Supplementary file 1 [file Table_1.DOCX]

Public Heath Empowerment Program Evaluation Questionnaire

**Gender: Male / Female**

**Age:**

1. **25-34**
2. **35-44**
3. **45-54**
4. **>=55**

**Highest educational degree earned (choose one only)**

**a. PhD**

**b. Master**

**c. Higher Diploma**

**d. Bachelor**

**e. Diploma**

**f. Other**

**Current Job title:**

**Place of working:**

**Country of graduation (Select one):**

1. **Pakistan**
2. **Sudan**
3. **Yemen**
4. **Jordan**
5. **Egypt**
6. **Iraq**
7. **Tunisia**
8. **KSA**
9. **Morocco**
10. **Other (Specify………)**

**Year of graduation:**

1. **PHEP version:**
2. **BFE**
3. **SPO**
4. **Nutrition**

**Please choose the most appropriate answer that applies for you:**

| **How often are you involved in the following activities?** | | | | | |
| --- | --- | --- | --- | --- | --- |
| **Activity** | **Frequency** | | | | |
| Conduct, review, and monitor surveillance data collection | Never | Rarely | sometimes | Often | Always |
| Perform descriptive data analysis | Never | Rarely | sometimes | Often | Always |
| Communicate information effectively with agency staff and with the local community | Never | Rarely | sometimes | Often | Always |
| Respond effectively to public health events, specifically, disease outbreaks | Never | Rarely | sometimes | Often | Always |
| Write a summary report on surveillance findings or an outbreak investigation | Never | Rarely | sometimes | Often | Always |
| Use Microsoft Excel or any software to enter, analyze, and display public health surveillance data | Never | Rarely | sometimes | Often | Always |
| Prepare and administer an oral presentation of their field work | Never | Rarely | sometimes | Often | Always |
| Monitor the global trends of COVID-19 and mortality through relevant websites | Never | Rarely | sometimes | Often | Always |
| Manage COVID-19 surveillance data (data analysis and reporting) | Never | Rarely | sometimes | Often | Always |
| Contribute to the development and distribution of a standard case definition for COVID-19 | Never | Rarely | sometimes | Often | Always |
| Provide support in applying isolation and infection control protocols for confirmed COVID-19 cases | Never | Rarely | sometimes | Often | Always |
| Collect samples and screen passengers for testing to confirm suspected COVID-19 cases | Never | Rarely | sometimes | Often | Always |
| Dissemination of health education messages and promotional materials to raise awareness towards COVID-19. | Never | Rarely | sometimes | Often | Always |
| Respond to public queries about COVID-19 through the specified hotlines and documents with standard appropriate information. | Never | Rarely | sometimes | Often | Always |
| Search for published scientific literature, standard operating procedures, and guidelines, and support development of the national guidelines for the COVID-19 epidemic. | Never | Rarely | sometimes | Often | Always |

| **How do you evaluate your skill in conducting this activity?** | | | | | |
| --- | --- | --- | --- | --- | --- |
| **Activity** | **Evaluation** | | | | |
| Conduct, review, and monitor surveillance data collection | Very Poor | poor | Acceptable | good | Very good |
| Perform descriptive data analysis | Very Poor | poor | Acceptable | good | Very good |
| Communicate information effectively with agency staff and with the local community | Very Poor | poor | Acceptable | good | Very good |
| Respond effectively to public health events, specifically, disease outbreaks | Very Poor | poor | Acceptable | good | Very good |
| Write a summary report on surveillance findings or an outbreak investigation | Very Poor | poor | Acceptable | good | Very good |
| Use Microsoft Excel or any software to enter, analyze, and display public health surveillance data | Very Poor | poor | Acceptable | good | Very good |
| Prepare and administer an oral presentation of their field work | Very Poor | poor | Acceptable | good | Very good |
| Monitor the global trends of COVID-19 and mortality through relevant websites | Very Poor | poor | Acceptable | good | Very good |
| Manage COVID-19 surveillance data (data analysis and reporting) | Very Poor | poor | Acceptable | good | Very good |
| Contribute to the development and distribution of a standard case definition for COVID-19 | Very Poor | poor | Acceptable | good | Very good |
| Provide support in applying isolation and infection control protocols for confirmed COVID-19 cases | Very Poor | poor | Acceptable | good | Very good |
| Collect samples and screen passengers for testing to confirm suspected COVID-19 cases | Very Poor | poor | Acceptable | good | Very good |
| Dissemination of health education messages and promotional materials to raise awareness towards COVID-19. | Very Poor | poor | Acceptable | good | Very good |
| Respond to public queries about COVID-19 through the specified hotlines and documents with standard appropriate information. | Very Poor | poor | Acceptable | good | Very good |
| Search for published scientific literature, standard operating procedures, and guidelines, and support development of the national guidelines for the COVID-19 epidemic. | Very Poor | poor | Acceptable | good | Very good |

| **How much the PHEP-BFE helped you to conduct this activity?** | | | | | |
| --- | --- | --- | --- | --- | --- |
| **Activity** | **Extent** | | | | |
| Conduct, review, and monitor surveillance data collection | Never | Little | Somewhat | Much | A Great Deal |
| Perform descriptive data analysis | Never | Little | Somewhat | Much | A Great Deal |
| Communicate information effectively with agency staff and with the local community | Never | Little | Somewhat | Much | A Great Deal |
| Respond effectively to public health events, specifically, disease outbreaks | Never | Little | Somewhat | Much | A Great Deal |
| Write a summary report on surveillance findings or an outbreak investigation | Never | Little | Somewhat | Much | A Great Deal |
| Use Microsoft Excel or any software to enter, analyze, and display public health surveillance data | Never | Little | Somewhat | Much | A Great Deal |
| Prepare and administer an oral presentation of their field work | Never | Little | Somewhat | Much | A Great Deal |
| Monitor the global trends of COVID-19 and mortality through relevant websites | Never | Little | Somewhat | Much | A Great Deal |
| Manage COVID-19 surveillance data (data analysis and reporting) | Never | Little | Somewhat | Much | A Great Deal |
| Contribute to the development and distribution of a standard case definition for COVID-19 | Never | Little | Somewhat | Much | A Great Deal |
| Provide support in applying isolation and infection control protocols for confirmed COVID-19 cases | Never | Little | Somewhat | Much | A Great Deal |
| Collect samples and screen passengers for testing to confirm suspected COVID-19 cases | Never | Little | Somewhat | Much | A Great Deal |
| Develop of health education messages and promotional materials to raise awareness towards COVID-19. | Never | Little | Somewhat | Much | A Great Deal |
| Respond to public queries about COVID-19 through the specified hotlines and documents with standard appropriate information. | Never | Little | Somewhat | Much | A Great Deal |
| Search for published scientific literature, standard operating procedures, and guidelines, and support development of the national guidelines for the COVID-19 epidemic. | Never | Little | Somewhat | Much | A Great Deal |
